# Myrislignan Exhibits Activities Against *Toxoplasma gondii* RH Strain by Triggering Mitochondrial Dysfunction

**DOI:** 10.3389/fmicb.2019.02152

**Published:** 2019-09-18

**Authors:** Jili Zhang, Hongfei Si, Bing Li, Xuzheng Zhou, Jiyu Zhang

**Affiliations:** ^1^Key Laboratory of Veterinary Pharmaceutical Development, Ministry of Agriculture, Lanzhou, China; ^2^Key Laboratory of New Animal Drug Project of Gansu Province, Lanzhou, China; ^3^Lanzhou Institute of Husbandry and Pharmaceutical Sciences, Chinese Academy of Agricultural Sciences, Lanzhou, China; ^4^Department of Medicinal Chemistry and Biology, College of Pharmacy, Jinan University, Guangzhou, China

**Keywords:** myrislignan, *T. gondii*, *in vitro*, *in vivo*, mechanism(s) of action

## Abstract

*Toxoplasma gondii* is a widespread obligatory parasitic protozoon that infects nearly all warm-blooded animals and causes toxoplasmosis. However, the current treatments for toxoplasmosis are limited by severe side effects. Myrislignan is a natural product from *Myristica fragrans* Houtt with wide pharmacological activities. In the current study, we tested the anti-*T. gondii* activity of myrislignan both *in vitro* and *in vivo* and explored its potential mechanism of action. The cytotoxicity of myrislignan in African green monkey kidney (Vero) cells was assessed using Cell Counting Kit-8 (CCK-8) assays. The *in vitro* effects of myrislignan on *T. gondii* were determined by quantitative PCR and Giemsa staining. An *in vivo* murine model of *T. gondii* infection was used to determine the efficacy of myrislignan. The changes in tachyzoites after myrislignan exposure were examined by electron microscopy. The impact of myrislignan on mitochondrial function in tachyzoites was assessed by MitoTracker Red CMXRos staining and an ATP detection kit. *In vitro*, myrislignan inhibited *T. gondii* tachyzoite proliferation with a 50% effective concentration of 32.41 μg/ml, and reduced the invasion of cells by tachyzoites (14.63 and 1.92% invasion rates for control and 70 μg/ml myrislignan, respectively). Importantly, myrislignan had no significant cytotoxicity against Vero cells at concentrations less than 132 μg/ml. In addition, surface shrinkage and mitochondrial damage were observed in tachyzoites after myrislignan exposure. The reduced Δ*Ψm* and ATP levels in tachyzoites treated with myrislignan further confirmed mitochondrial damage. In the *in vivo* murine model, myrislignan treatment significantly reduced the parasite burden in tissues compared to no treatment. In conclusion, myrislignan had potent anti-*T. gondii* activities both *in vitro* and *in vivo,* and these activities might involve the interruption of mitochondrial function. These data suggest that myrislignan might be a useful compound for the treatment of toxoplasmosis.

## Introduction

*Toxoplasma gondii,* an obligate intracellular apicomplexan parasite, causes toxoplasmosis in most warm-blooded animals, including humans (Weiss and Kim, 2013). *T. gondii* rarely causes severe symptoms in healthy individuals, but it can develop into life-threatening toxoplasmic encephalitis from primary or recrudescent infections in immunocompromised patients (Machala et al., 2015). Importantly, *T. gondii* infections during pregnancy can result in vertical transmission, which has severe consequences for the fetus, including fetal malformations, intracranial calcifications, mental retardation, hydrocephalus, impaired visual acuity, and even death (Fallahi et al., 2018).

Currently, the treatment drugs for toxoplasmosis include clindamycin, azithromycin, sulfadiazine, pyrimethamine, and atovaquone. To date, the combination of pyrimethamine and sulfadiazine is considered as the frontline treatment for toxoplasmosis (Giovati et al., 2018; Munera López et al., 2019). However, this treatment has several side effects (de-la-Torre et al., 2011), such as bone marrow arrest, hypersensitive reactions, agranulocytosis and megaloblastic anemia (Rothova et al., 1993; Bosch-Driessen et al., 2002; Guaraldo et al., 2018). Thus, new drugs with lower toxicity for the treatment of this infection are urgently needed.

In recent years, natural products found in plants have become a useful source of drugs for clinical use (Sepulveda-Arias et al., 2014). *Myristica fragrans* Houtt (Myristicaceae) is an aromatic evergreen tree, considered as the Spice Islands (van Gils and Cox, 1994), and its seeds, which are commonly known as nutmeg, are not only a spice for cooking but also a medicine with a wide range of pharmacological activities. Myrislignan is the main active ingredient of nutmeg (Isogai et al., 1973) and has various biological activities, including anti-inflammatory activity (Jin et al., 2012), anti-cancer activity (Lu et al., 2017), and nitric oxide production inhibitory activity (Cao et al., 2015). Herein, we investigated the activity of myrislignan against the *T. gondii* RH strain and preliminarily explored the underlying mechanism of action.

## Materials and Methods

### Ethics

BALB/c mice (8 weeks, 18–20 g, female) were kept in cages with an adequate temperature (25 ± 2°C) and provided water and food. All experiments were approved by the Animal Administration and Ethics Committee of Lanzhou Institute of Husbandry and Pharmaceutical Sciences, Chinese Academy of Agricultural Sciences. The certificate number was SCXK (Gan) 2014-0002. All procedures in this study were strictly carried out accordance with good laboratory animal practice standards according to the Animal Ethics Procedures and Guidelines of the People’s Republic of China. All efforts were made to minimize animal suffering.

### Cells

African green monkey kidney (Vero) cells were kindly provided by the Chinese Academy of Sciences (Shanghai, China). Cells were cultured in Dulbecco’s Modified Eagle’s Medium (DMEM, Gibco, USA) with penicillin (100 U/ml, Gibco, USA), streptomycin (100 μg/ml, Gibco, USA), 1% non-essential amino acids (NEAAs, Gibco, USA), 1% GlutaMAX (Gibco, USA), and 10% heat-inactivated fetal bovine serum (FBS, Gibco, USA) at 37°C in an atmosphere with 5% CO_2_.

### Drugs

Myrislignan ([erythro-(1R,2S)-2-(4-allyl-2,6-dimethoxyphenoxyl)-1-(4-hydroxy-3-methoxyphenyl) propan-1-ol], batch number DST180502-043) was purchased from Desite Biotechnology Co., Ltd. (Chengdu, China) and had a purity greater than 99%. For the *in vitro* experiments, myrislignan was dissolved in dimethyl sulfoxide (DMSO, Sigma, USA) to a concentration of 25 mg/ml and diluted in DMEM containing 1% FBS to different concentrations. Sulfadiazine (Sigma, USA), which was used as positive control drug, was dissolved in DMEM with 1% FBS to a concentration of 0.4 μg/ml. For the *in vivo* experiments, myrislignan was dissolved in solvent 1 (isotonic saline containing 5% ethanol and 5% Cremophor EL) to concentrations of 5 or 10 mg/ml. Sulfadiazine (10 mg/ml), pyrimethamine (5 mg/ml, Sigma, USA), and folinic acid (1.5 mg/ml, Sigma, USA) were suspended in physiological saline containing 1% CMC-Na (solvent 2) and used as a positive control.

### Parasites

*T. gondii* RH strain tachyzoites used in our study were obtained from Lanzhou Veterinary Research Institute, China. The tachyzoites were maintained in Vero cells in DMEM supplemented with 1% FBS and collected by the cell monolayer trypsinization and centrifugation of the suspensions for 10 min at 1500 ×*g*. The tachyzoites were released through a 27-gauge needle, and filtered through polycarbonate membrane filters with 5-μm pores to separate the tachyzoites from the host cells. Then, the samples were centrifuged for another 10 min at 1,500 ×*g* and resuspended in DMEM containing 1% FBS. The number of tachyzoites was determined by counting with a hemocytometer under a light microscope. The *T. gondii* infection experiments were performed under biosafety level 2 (BSL-2) conditions to ensure that the technicians and researchers who worked with this highly virulent strain of *T. gondii* (RH) were not infected.

### Cytotoxicity Assay

Vero cells were plated in 96-well plates (1 × 10^5^ cells/ml, 100 μl/well) in a monolayer and incubated with various concentrations of myrislignan in DMEM with 1% FBS or cultured in DMEM without myrislignan as a control. After 24 h, 10 μl of Cell Counting Kit-8 (CCK-8) (Biomake, USA) solution in DMEM was added to each well and then incubated for 2 h at 37°C and 5% CO_2_. The absorbance values were then measured at 450 nm using a Multiskan GO instrument (Thermo Fisher Scientific, MA, USA). The cell viability values are expressed as percentages of the control value (considered as 100% survival).

### Anti-proliferation Effect of Myrislignan on *T. gondii*

The anti-proliferation effect of myrislignan on *T. gondii* tachyzoites was determined by real-time quantitative PCR (qPCR) (Zhang et al., 2019). Confluent Vero monolayers in six-well plates were infected with 2.5 × 10^4^ fresh tachyzoites per well for 6 h, the cells were washed twice with PBS to remove extracellular tachyzoites and then incubated with DMEM (1% FBS) containing various concentrations of myrislignan (20, 30, 40, 50, 60, 70, 80, or 90 μg/ml) at 37°C for 24 h. Sulfadiazine (0.4 μg/ml) was added as a positive drug control under identical conditions. Infected Vero cells incubated with DMEM (1% FBS) without any drug exposure served as the parasite control group, and uninfected Vero cells cultured in DMEM (1% FBS) served as the host cell control group. After 24 h of incubation, total genomic DNA from each well was isolated using DNAiso Reagent (Takara), and the 529-bp repeat element of *T. gondii* was measured by qPCR (Homan et al., 2000). PCR amplification was performed using a QuantStudio 6 Flex Real-Time PCR System (Life Technologies), as described previously (Si et al., 2018). The number of tachyzoites was calculated according to the standard curve (*Y* = −0.1979*x* + 9.9926; *R*^2^ = 0.9945), which was constructed using DNA samples obtained from the RH strain tachyzoites at a range of serial dilutions (2.81 × 10^6^ to 5.49× 10^3^/ml). No amplification product was observed in the host cell control wells. The 50% effective concentration (EC_50_) was calculated. For each experimental group, the qPCR assay was performed in independent triplicates.

### Anti-invasion Activity of Myrislignan on *T. gondii*

Vero cell monolayers in six-well plates were cultured in DMEM (1% FBS) containing myrislignan (20, 30, 40, 50, 60, or 70 μg/ml), sulfadiazine (0.4 μg/ml), or no drug (as a parasite control) and then infected with 2.5 × 10^6^ fresh tachyzoites per well. After 2 h of infection, Vero cell monolayers were stained with Giemsa staining, as previously described (Zhang et al., 2019). The number of infected cells was counted in at least 15 random fields per well using 40× objective lens light microscopy. The percentage of invasion was expressed as the number of infected cells compared with the total number of host cells. Triplicate independent experiments were performed.

### Dose Safety Assessment *in vivo*

Preliminary experiments were performed to determine safe doses of myrislignan. Mice were divided into four groups (6 mice/group) as follows: myrislignan (50 or 100 mg/kg, intraperitoneal injection, twice a day), positive drug (100 mg/kg sulfadiazine, 50 mg/kg pyrimethamine, and 15 mg/kg folinic acid, oral administration, once a day), and PBS (intraperitoneal injection, twice a day). Treatments lasted for 30 days. The body weights, body temperatures, and clinical symptoms were monitored daily.

### Parasite Burden in the Tissues of Infected Mice

To evaluate the therapeutic effect of myrislignan on acute infection, mice were infected intraperitoneally with 1 × 10^4^ tachyzoites. After 4 h of infection, the mice were divided into three groups (6 mice/group) for treatment with PBS (as a parasite control), myrislignan (100 mg/kg), or positive control drug, as described above in Section “Dose Safety Assessment *in vivo*.” The treatments were administered for 4 consecutive days. Then, the mice were humanely killed by cervical dislocation, and the brains, livers, and spleens were removed and homogenized in PBS. Total genomic DNA was isolated from the tissue homogenates using tissue DNA kits (D3396, Omega, Omega Bio-Tek, USA). The *T. gondii* 529-bp gene was quantified by qPCR as described above in Section “Anti-proliferation Effect of Myrislignan on *T. gondii*.” The number of parasites in the tissue samples was deduced from standard curves constructed using known numbers of tachyzoites.

### Electron Microscopy Analysis

For scanning electron microscopy (SEM) analysis, Vero cells were seeded on coverslips and infected with 5 × 10^4^ tachyzoites per well. Eight hours after infection, myrislignan was added at a concentration of 32 or 70 μg/ml, and cultured for an additional 8 or 24 h. Vero cells were then fixed with glutaraldehyde for 1.5 h and washed with PBS (pH = 7.4) to remove the glutaraldehyde. The samples were fixed with 1% osmium acid solution for 1.5 h, washed with PBS to remove the osmium acid, and dehydrated in a graded alcohol series. The cells were subjected to critical point drying, and the upper portion of the cells was removed with adhesive tape, revealing the internal organization of the parasitophorous vacuoles (PVs) (Si et al., 2018). The Vero cells were then sputter-coated with gold-palladium and observed under a scanning electron microscope (Nova Nano SEM 450, Thermo, FEI, USA).

For transmission electron microscopy (TEM) analysis, Vero cells were grown to confluence in T25 flasks and infected with 1.5 × 10^6^ *T. gondii* tachyzoites for 8 h. After 32 or 70 μg/ml myrislignan was added, the cells were cultured for 8 or 24 h, digested with TrypLE Express for 2 min, washed twice with PBS. Then, the cells were processed for transmission electron microscopy (TEM, Tecnai Spirit Bio-TWIN, Thermo, FEI, USA) as described previously (Si et al., 2018).

### Evaluation of the Effect of Myrislignan on *T. gondii* Tachyzoites in Mitochondria

Extracellular parasites (1 × 10^5^ per group) collected as described in Section “Parasites” were maintained in suspension with myrislignan (32 or 70 μg/ml) in DMEM or without any drug (positive or negative control) for 8 h at 37°C. The negative control samples were incubated for 20 min with 10 μM carbonyl cyanide 3-chlorophenylhydrazone (CCCP), which can cause Δ*Ψm* depletion. After incubation, the tachyzoite samples were stained with the MitoTracker Red CMXRos probe (50 nM, Invitrogen, USA) for 20 min, as described previously with some modifications (Besteiro et al., 2011).

Vero cells were placed in cell culture dishes and incubated with myrislignan (70 μg/ml) or without any drug (as a positive control) in DMEM for 8 h at 37°C, stained with the MitoTracker Red CMXRos probe (250 nM) for 20 min, rinsed twice with PBS, fixed with 4% polyformaldehyde for 15 min, and washed twice with PBS to remove the polyformaldehyde, stained with Hoechst 33342 (C1027, Beyotime Institute of Biotechnology, China). The fluorescence intensity was observed by laser confocal microscopy. The relative fluorescence intensity of the cells was measured using an Enspire Microplate Reader (PerkinElmer, German).

### Measurement of the Adenosine Triphosphate Level

Fresh extracellular parasites (1 × 10^6^ per group) were collected and incubated with myrislignan (32 or 70 μg/ml) in DMEM or without any drug as positive or negative control. The negative control samples were incubated with CCCP, as described above in section “Evaluation of the Effect of Myrislignan on *T. gondii* Tachyzoites in Mitochondria.” After 8 h of incubation, the adenosine triphosphate (ATP) concentration in RH *T. gondii* tachyzoites in each group was quantified using a luciferase-based enhanced ATP assay kit (S0027, Beyotime Institute of Biotechnology, China), as previously described (Zhang et al., 2019). The luminescence of each well was detected using a multilabel reader (EnSpire, PerkinElmer, USA). The ATP levels were presented as a percentage of positive control group ATP levels. Triplicate independent experiments were performed.

### Statistical Analyses

The IC_50_ of myrislignan on Vero cell growth and the EC_50_ of myrislignan on tachyzoite growth inhibition were plotted as functions of the drug concentration through a nonlinear curve analysis using SPSS 19.0 software (SPSS Inc., Chicago, IL, USA). Data comparisons between different groups were statistically analyzed by one-way analysis of variance (ANOVA) using SPSS software. Differences were considered statistically significant at a *p* value <0.01.

## Results

### Myrislignan Inhibited *T. gondii* Tachyzoite Intracellular Proliferation and Invasion *in vitro*

**Figure 1:**
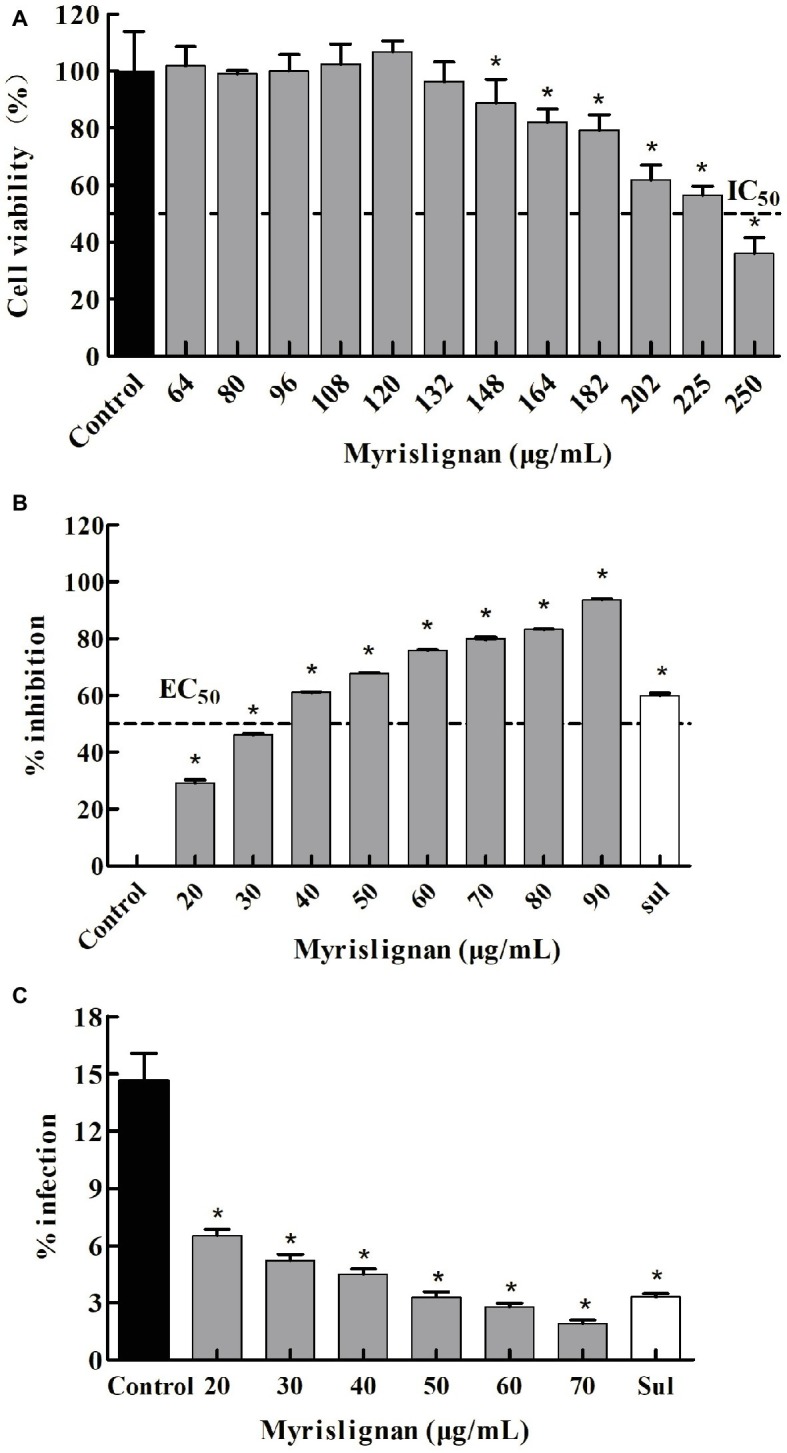
Myrislignan cytotoxicity in Vero cells **(A)**. Vero cells were cultured with myrislignan (64, 80, 96, 108, 120, 132, 148, 164, 182, 202, 225, or 250 μg/ml) or without (control) for 24 h, and the percentages of viable cells were quantified *via* a CCK-8 assay. All data are presented as the mean ± standard deviation (SD), and the experiments were performed in triplicate. ^*^p < 0.01 compared with the control. Assessment of the anti-proliferation effect of myrislignan on *T. gondii*-infected Vero cells by qPCR **(B)**. Vero cells were infected with 2.5 × 10^4^ *T. gondii* for 6 h and then treated with myrislignan (20, 30, 40, 50, 60, 70, 80, or 90 μg/ml), sulfadiazine (0.4 μg/ml) or no drug (parasite control). After 24 h of treatment, total *T. gondii* DNA in each group was extracted, and the 529-bp repeat element of the *T. gondii* genome was measured by PCR. The percent inhibition of tachyzoite proliferation in each group was compared with that in the parasite control group. The data are presented as the mean ± SD of three independent experiments. ^*^p < 0.01 compared with the parasite control. Effects of myrislignan on *T. gondii* invasion **(C)**. Vero cells were cultured in DMEM with myrislignan (20, 30, 40, 50, 60, or 70 μg/ml), sulfadiazine (0.4 μg/ml), or no drug (parasite control) and infected with 2.5 × 10^6^ *T. gondii* tachyzoites. After 2 h of infection, the cells were subjected to Giemsa staining, and the percentage of infected cells in each group was determined by light microscopy. At least 15 random fields per well were counted. Triplicate independent experiments were performed, and the data are presented as the mean ± SD. ^*^p < 0.01 compared with the parasite control.

In the preliminarily cytotoxicity assay, myrislignan had no toxicity on the growth of Vero cells at concentrations lower than 132 μg/ml (Figure 1A). The IC_50_ value of myrislignan on Vero cell growth was 228.22 μg/ml. The final concentrations of DMSO in the cultures did not exceed 1% (v/v) and had no effect on cell proliferation. These assays established a safe dose range of myrislignan for subsequent tests.

The activity of myrislignan against the intracellular replication of tachyzoites within Vero cells was observed. In addition, a negative correction between the number of tachyzoites and myrislignan concentration was defined. According to the qPCR results, an increased myrislignan concentration (at 20–90 μg/ml) caused a significant (*p* < 0.01) reduction in the intracellular replication of *T. gondii* tachyzoites in a concentration-dependent manner (Figure 1B). The EC_50_ of myrislignan for tachyzoite growth inhibition was 32.41 μg/ml, which was higher than that of the positive control drug (sulfadiazine, 0.4 μg/ml), under identical experimental conditions.

Myrislignan treatment also showed significant inhibition of *T. gondii* invasion compared with that found in the parasite control group (1.92 and 14.63% invasion rates for 70 μg/ml myrislignan and the control treatment, respectively; *p* < 0.01) (Figure 1C). The positive drug control group exhibited 3.30% invasion.

### Myrislignan Exhibited Anti-*T. gondii* Activity *in vivo*

**Figure 2:**
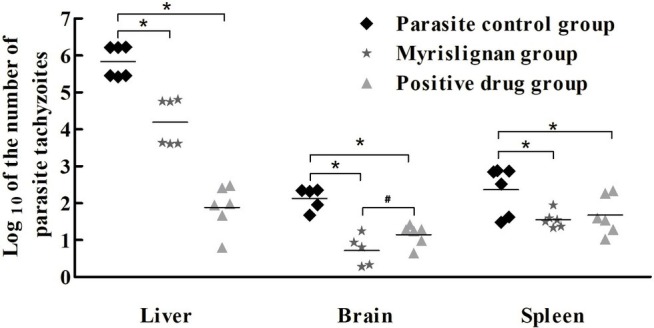
Myrislignan treatment reduced the parasite burden in the tissues of acutely infected mice. The mice were infected intraperitoneally with 1 × 10^4^ *T. gondii* tachyzoites and treated with myrislignan (100 mg/kg·bw), positive control drug or PBS (parasite control) for 4 days. The brains, livers, and spleens of the infected BALB/c mice were isolated and homogenized. Total genomic DNA was isolated, and the *T. gondii* 529-bp gene was detected by qPCR. The quantified parasite loads in the mouse brains, livers and spleens are presented as the log10 values of the numbers of tachyzoites per 20 mg of tissue. ^*^p < 0.01 compared with the parasite control; ^#^*p* < 0.01 compared with the positive control drug group.

Myrislignan at 50 or 100 mg/kg ip, bid, the positive control drug and PBS were demonstrated to be safe in healthy mice, and no clinical toxicity syndrome was observed during the 30-day treatment. Myrislignan and the positive control drug treatments significantly (*p* < 0.01) reduced the parasite load in the brain, liver, and spleen tissues compared with that found in the parasite control untreated group (Figure 2). Interestingly, myrislignan treatment was more effective at decreasing the parasite load in brains of infected mice than positive control drug treatment (*p* < 0.01).

### Myrislignan Induced Ultrastructural Changes in *T. gondii* Tachyzoites

**Figure 3:**
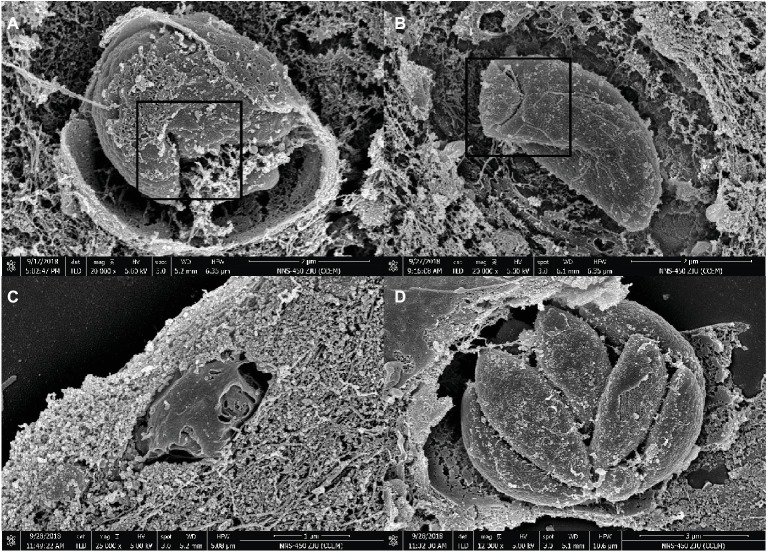
SEM micrograph of Vero cells infected with *T. gondii* and treated or not with myrislignan (32 or 70 μg/ml). Vero cells were infected with 5 × 10^4^ tachyzoites per well. After 8 h, myrislignan was added at a concentration of 32 μg/ml or 70 μg/ml. The cells were cultured for an additional 8 h or 24 h. After 8 h of treatment with myrislignan, the characteristic organization of the tachyzoites was distorted, as shown in **(A,B)**. After 24 h of treatment with myrislignan, the tachyzoites became atrophied, and their surface structure was lost, as shown in **(C)**. However, the organization of the parasites with uniform size and smooth surfaces into cysts and proliferation in a banana-like structure is shown in **(D)**. Scale bars: 2 μm **(A,B)**; 1 μm **(C)**; 3 μm **(D)**.

As demonstrated by SEM, the untreated parasite control group showed similar sizes and smooth surfaces organized into PVs, and their proliferation resulted in a banana-like structure (Figure 3D). However, treatment with myrislignan at 32 or 70 μg/ml for 8 h affected the characteristic organization of tachyzoites inside the PVs (Figures 3A,B), and at 24 h of treatment, the tachyzoites showed surface shrinkage and lost their typical structure (Figure 3C).

**Figure 4:**
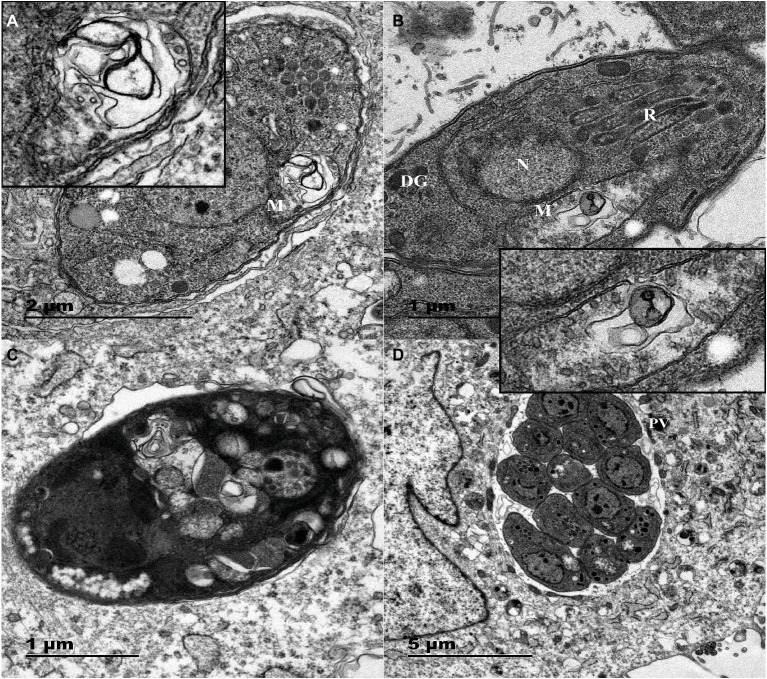
TEM images of Vero cells infected with *T. gondii* and treated or not with myrislignan (32 or 70 μg/ml). Vero cells in T25 flasks were infected with 1.5 × 10^6^ *T. gondii* tachyzoites for 8 h; then, 32 μg/ml or 70 μg/ml myrislignan was added, the cells were cultured for 8 or 24 h. The nucleus (N), dense granules (DGs), rhoptries (Rs), and mitochondrion (M) in these tachyzoites were clearly detected, 8 h of treatment with myrislignan (32 or 70 μg/ml) induced changes in the mitochondria of tachyzoites, as shown in **(A)** and **(B)**. These changes included the appearance of swelling and deformation as well as destruction of the ridge structure. After 24 h of treatment with myrislignan, the cytoplasmic structure and PV membranes of the tachyzoites had completely disappeared, and the parasites exhibited progressive degeneration **(C)**. However, in the typical morphology of parasites incubated in the absence of myrislignan, the parasites were undergoing proliferation by endodyogeny within the PV **(D)**. Scale bars: 1 μm **(B,C)**; 2 μm **(A)**; 5 μm **(D)**.

The TEM results confirmed that the untreated parasites displayed a well-preserved intracellular space with typical apicomplexan structural features, and the parasites proliferated by endodyogeny within the PVs surrounded by an obvious PV membrane, as shown in Figure 4D. However, myrislignan treatment for 8 h induced mitochondrial swelling and deformation as well as destruction of the ridge structure in *T. gondii*, as shown in Figures 4A,B. Interestingly, no significant changes were observed in other organelles. In addition, at 24 h of treatment with myrislignan, the cytoplasmic structure and PV membranes of tachyzoites had completely disappeared, and progressive degeneration of the parasites was observed, as shown in Figure 4C.

### Myrislignan Induced Mitochondrial Damage in *T. gondii*

**Figure 5:**
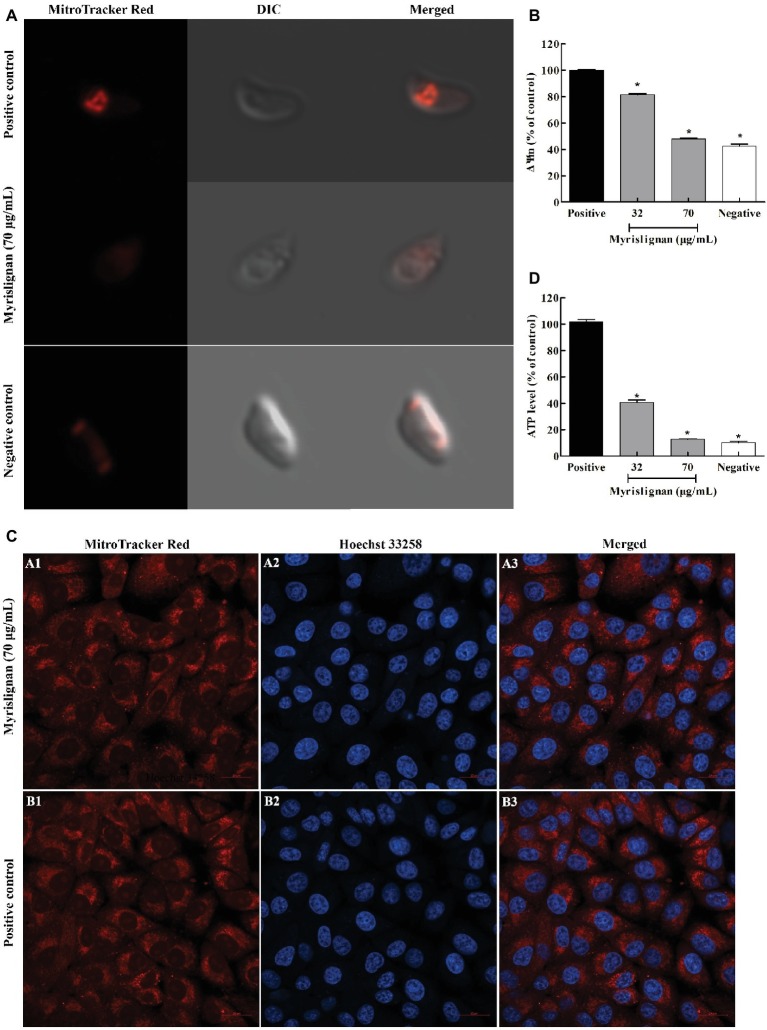
Myrislignan decreased the Δ*Ψm* and ATP levels of *T. gondii* tachyzoites. *T. gondii* tachyzoites (1 × 10^5^ per group) were incubated with myrislignan (32 or 70 μg/ml) or with no drug (positive or negative control) in DMEM for 8 h at 37°C. The negative control samples were further incubated with 10 μM CCCP for 20 min. All the samples were then stained with MitoTracker Red CMXRos for 20 min at 37°C in the dark, rinsed twice with PBS, centrifuged and suspended in 500 μl of PBS. The fluorescence intensity of each group was then observed by laser confocal microscopy **(A)**. The fluorescence intensities of the positive or negative control group and 32 μg/ml and 70 μg/ml myrislignan treatment groups were assessed using a multilabel reader **(B)**. Evaluation of the effect of myrislignan on the mitochondria of Vero cells **(C)**. Vero cells were placed in cell culture dishes and incubated with myrislignan (70 μg/ml) in DMEM or without drug (positive control) for 8 h at 37°C, stained with the MitoTracker Red CMXRos probe (250 nM) for 20 min at 37°C, rinsed twice with PBS, fixed with 4% polyformaldehyde for 15 min, washed twice with PBS to remove the polyformaldehyde, stained with Hoechst 33342 and then observed by laser confocal microscopy. Myrislignan induced a decrease in the ATP concentration **(D)**. *T. gondii* tachyzoites (1 × 10^6^ per group) were incubated with myrislignan (32 or 70 μg/ml) or without any drug (parasite positive or negative control) in DMEM for 8 h at 37°C. The negative control samples were then incubated with CCCP. The ATP levels of *T. gondii* tachyzoites were determined using a multilabel reader. The ATP levels are expressed as a percentage of the positive control. Data are presented as the mean value ± SD from three replicate experiments. ^*^p < 0.01 compared with the positive parasite control.

A MitoTracker Red CMXRos probe was used to evaluate the possible mitochondrial damage in tachyzoites after myrislignan treatment. We found that exposure of *T. gondii* to myrislignan for 8 h resulted in significant dose-dependent decreases in the Δ*Ψm* of *T. gondii* (*p* < 0.01; Figures 5A,B). However, myrislignan treatment did not affect the Δ*Ψm* in Vero cells (Figure 5C).

To further explore whether the effects of myrislignan on *T. gondii* are associated with energy changes, the ATP content was measured. Eight hours of treatment with myrislignan induced significant dose-dependent decreases in the ATP levels in extracellular parasites compared with the conditions in the positive control group (*p* < 0.01; Figure 5D).

## Discussion

Recent years, many studies have focused on finding safe drugs with novel mechanisms of action against *T. gondii*, and some of these novel compounds may represent good starting points for the discovery of effective new drugs (Montazeri et al., 2017). Myrislignan, a main active ingredient of nutmeg, is known to exhibit various bioactivities, such as affecting the enzymatic activity of hepatic mixed-function oxidase (Shin and Woo, 1990) and protecting against thioacetamide-induced liver injury (Yang et al., 2018). The present study investigated whether myrislignan exhibits anti-*T. gondii* activity both *in vitro* and in an animal model and its mechanisms of action. To the best of our knowledge, we demonstrated for the first time that myrislignan had potent anti-*T. gondii* activity with no significant cytotoxic effects on Vero cells at concentrations lower than 132 μg/ml. Myrislignan inhibited intracellular replication with an EC_50_ of 32.41 μg/ml and reduced the invasion of *T. gondii* tachyzoites in a concentration-dependent manner. A previous study that reported nutmeg essential oil inhibits anti-*T. gondii* activity with an EC_50_ value of 24.45 μg/ml, which is similar to the value of myrislignan in our results, but the IC_50_ of nutmeg essential oil in Vero cells was 24.83 μg/ml (Pillai et al., 2012). In comparison, myrislignan has a wider safety range, according to its IC_50_ of 228.22 μg/ml. Previous studies have also reported that myrislignan exhibits cytotoxicity toward several cancer cells, but did not affect macrophage cell viability at doses ranging from 6.25 to 50 μg/ml (Jin et al., 2012; Lu et al., 2017). These *in vitro* results suggest that myrislignan exhibits potent anti-*T. gondii* activity *in vitro* in the absence of host toxicity.

Subsequently, a mouse model was established by infecting mice with the virulent RH strain of *T. gondii* to determine whether myrislignan exerts anti-*T. gondii* effects on acute infections *in vivo.* Using this model, we found that myrislignan significantly reduced the parasite burden in tissues, especially in brain tissues. In addition, our previous study also showed that myrislignan can protect 40% of acute infected mice from death (Supplementary Figure S1). Notably, myrislignan can penetrate the blood-brain barrier, which serves as the primary interface between the central nervous system and the systemic circulation (Wu et al., 2016). This property may contribute to the treatment effect of myrislignan against *T. gondii in vivo*. Furthermore, clinical toxicity syndrome was not observed upon myrislignan treatment in healthy mice. Therefore, our findings indicate that myrislignan exerts anti-*T. gondii* effects on acute infections *in vivo*.

A previous study revealed that myrislignan induces apoptosis and cell cycle arrest in A549 cells by activating mitogen-activated protein kinase, inhibiting the epidermal growth factor receptor signaling pathway, changing the Δ*Ψm*, releasing c-Myc, and decreasing the anti-apoptosis level (Lu et al., 2017). Herein, the SEM analysis revealed that myrislignan treatment induced a change in the typical surface structure of *T. gondii* tachyzoites, and the TEM analysis showed mitochondrial damage. Accordingly, these results indicated that myrislignan treatment against *T. gondii* might interfere with *T. gondii* mitochondrial function. Δ*Ψm* is an important indicator of normal mitochondrial configuration and function. MitoTracker Red CMXRos staining after myrislignan treatment confirmed that the Δ*Ψm* of tachyzoites was markedly decreased, suggesting mitochondrial damage. Furthermore, mitochondria are the center of energy metabolism, and mitochondrial damage may cause changes in ATP levels. Our results showed that myrislignan treatment also induced dose-dependent decreases in the ATP levels in *T. gondii*. Therefore, these emerging lines of evidence support the notion that the mechanism of action of myrislignan against *T. gondii* might involve interference with *T. gondii* mitochondrial function. As mentioned above, myrislignan induces apoptosis and cell cycle arrest in A549 cells and changes the Δ*Ψm* (Lu et al., 2017). However, myrislignan did not induce mitochondrial changes in host cells. In addition, unlike mammalian cells, *T. gondii* tachyzoites have only one mitochondrion (Melo et al., 2000); thus, mitochondrial damage may lead to an inadequate energy supply in *T. gondii* tachyzoites and eventually death. However, the specific mechanism of action needs to be further explored.

In conclusion, the findings suggest that myrislignan exerts anti-*T. gondii* activity by inhibiting its replication and invasion *in vitro* in the absence of host toxicity. Myrislignan reduces the parasite burden in the tissues of infected mice. The mechanism of action of myrislignan against *T. gondii* might be associated with *T. gondii* mitochondrial function. Therefore, our work suggested that myrislignan has the potential to be developed into a novel anti-*Toxoplasma* agent, but further investigations are needed to evaluate the therapeutic efficacy of myrislignan against different *T. gondii* strains and to explore its specific anti-*T. gondii* mechanism of action.

## Data Availability

All datasets for this study are included in the manuscript/supplementary files.

## Ethics Statement

All experiments were approved by the Animal Administration and Ethics Committee of Lanzhou Institute of Husbandry and Pharmaceutical Sciences, Chinese Academy of Agricultural Sciences. The certificate number was SCXK (Gan) 2014-0002. All procedures in this study were carried out strictly in accordance with good laboratory animal practice standards according to the Animal Ethics Procedures and Guidelines of the People’s Republic of China. All efforts were made to minimize animal suffering.

## Author Contributions

HS, XZ, and BL revised the manuscript. JiyZ directed the project and reviewed the manuscript. JilZ supervised the experiments and wrote the manuscript. All authors read and approved the final manuscript.

### Conflict of Interest Statement

The authors declare that the research was conducted in the absence of any commercial or financial relationships that could be construed as a potential conflict of interest.

## Supplementary Material

Supplementary Figure S1 Effect of myrislignan on the survival rate of acutely infected mice. Mice into 4 groups consisting of 10 mice each and injected 100 tachyzoites intraperitoneally into each mouse. After 4 h, mice were treated with myrislignan (50 or 100 mg/kg·bw, intraperitoneal injection, twice a day), the positive drugs (100 mg/kg·bw sulfadiazine, 50 mg/kg·bw pyrimethamine or 15 mg/kg·bw folinic acid, oral administration, once a day), PBS (parasite control group, intraperitoneal injection, twice a day), respectively. The treatments were administered for 7 consecutive days, and the mice were observed for 8 days after treatment cessation. The survival times of the infected mice and the numbers of deaths were monitored and recorded twice daily for 15 days.
