# Supplementary figures and images for: Myrislignan Exhibits Activities Against Toxoplasma gondii RH Strain by Triggering Mitochondrial Dysfunction
Source: Front Microbiol. 2019 Sep 18;10:2152. doi: 10.3389/fmicb.2019.02152 (PMC6759950; doi:10.3389/fmicb.2019.02152)

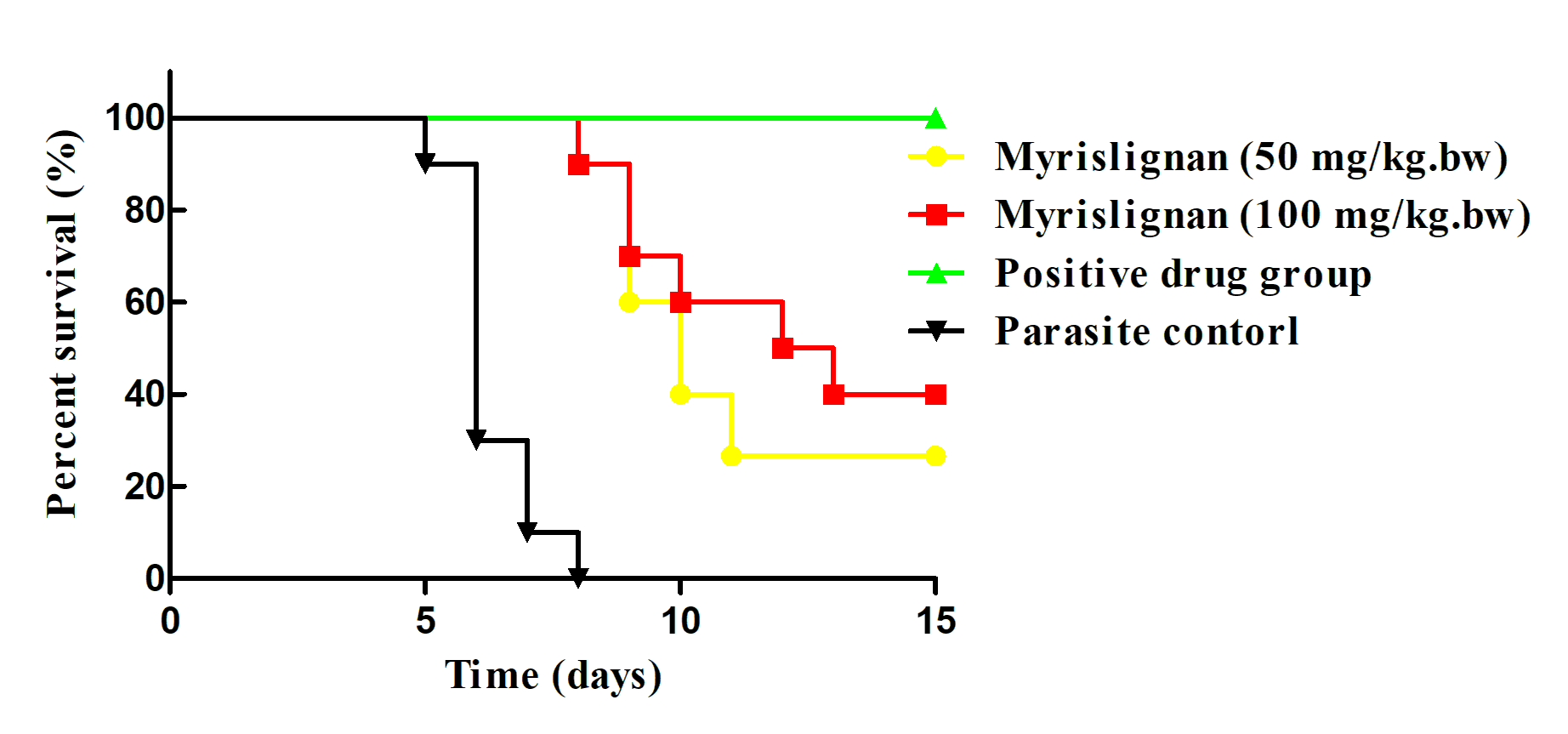

Supplement: Supplementary Figure S1 — Effect of myrislignan on the survival rate of acutely infected mice. Mice into 4 groups consisting of 10 mice each and injected 100 tachyzoites intraperitoneally into each mouse. After 4 h, mice were treated with myrislignan (50 or 100 mg/kg·bw, intraperitoneal injection, twice a day), the positive drugs (100 mg/kg·bw sulfadiazine, 50 mg/kg·bw pyrimethamine or 15 mg/kg·bw folinic acid, oral administration, once a day), PBS (parasite control group, intraperitoneal injection, twice a day), respectively. The treatments were administered for 7 consecutive days, and the mice were observed for 8 days after treatment cessation. The survival times of the infected mice and the numbers of deaths were monitored and recorded twice daily for 15 days. [file Image_1.TIF]
